# Association between infertility and incident onset of systemic autoimmune rheumatic disease after childbirth: a population-based cohort study

**DOI:** 10.1093/humrep/deae253

**Published:** 2024-12-05

**Authors:** Natalie V Scime, Maria P Velez, May Y Choi, Joel G Ray, Alexa Boblitz, Hilary K Brown

**Affiliations:** Department of Health and Society, University of Toronto Scarborough, Toronto, ON, Canada; ICES, Toronto, ON, Canada; ICES, Toronto, ON, Canada; Department of Obstetrics and Gynecology, Queen’s University, Kingston, ON, Canada; Cumming School of Medicine, University of Calgary, Calgary, AB, Canada; ICES, Toronto, ON, Canada; Dalla Lana School of Public Health, University of Toronto, Toronto, ON, Canada; Li Ka Shing Knowledge Institute, St. Michael’s Hospital, Toronto, ON, Canada; ICES, Toronto, ON, Canada; Department of Health and Society, University of Toronto Scarborough, Toronto, ON, Canada; ICES, Toronto, ON, Canada; Dalla Lana School of Public Health, University of Toronto, Toronto, ON, Canada

**Keywords:** infertility, autoimmune diseases, cohort studies, mothers, female, Ontario

## Abstract

**STUDY QUESTION:**

What is the association between infertility with or without fertility treatment and incident onset of systemic autoimmune rheumatic disease (SARD) among women who give birth?

**SUMMARY ANSWER:**

Women who experienced infertility but did not use fertility treatment had a higher incidence of SARD up to 9 years after delivery than those who did not experience infertility, even after accounting for their higher rates of preeclampsia, spontaneous preterm birth, and stillbirth.

**WHAT IS KNOWN ALREADY:**

Infertility is increasingly common and is an under-appreciated risk marker for chronic diseases in women. Despite several studies documenting abnormal immune activity in women with infertility, little is known about the association between infertility and incidence of autoimmune diseases such as SARD which disproportionately develops in reproductive-aged women.

**STUDY DESIGN, SIZE, DURATION:**

This population-based cohort study using linked administrative data for all of ON, Canada, 2012–2021 and included 568 053 singleton births among 465 078 women aged 18–50 years without known pre-existing SARD.

**PARTICIPANTS/MATERIALS, SETTING, METHODS:**

The exposures were: (i) no infertility with unassisted conception (referent [88.0% of the cohort]); (ii) infertility without fertility treatment (9.2%); (iii) infertility with non-invasive fertility treatment (ovulation induction or intrauterine insemination [1.4%]); and (iv) infertility with invasive fertility treatment (IVF or ICSI [1.4%]). SARD was identified by a validated algorithm based on diagnostic codes at two physician visits, one rheumatologist visit, or one hospitalization and measured from the index delivery date, with censoring at death, loss of health insurance, or study end of 31 March 2021. Marginal structural Cox proportional hazards models generated hazard ratios (HR) and 95% CIs representing total effects adjusted for sociodemographic characteristics, comorbidities, and smoking, and controlled direct effects additionally accounting for adverse pregnancy outcomes.

**MAIN RESULTS AND THE ROLE OF CHANCE:**

The median (IQR) duration of follow-up was 6.5 (4–9) years. The incidence rate of SARD was 9.3 per 10 000 person-years in women without infertility, 12.5 per 10 000 person-years in those with infertility and no fertility treatment, 10.9 per 10 000 person-years following non-invasive fertility treatment, and 10.9 per 10 000 person-years after invasive fertility treatment. Infertility without treatment was associated with an elevated risk of SARD, even after accounting for adverse pregnancy outcomes (controlled direct effect HR 1.25, 95% CI 1.12–1.40). Neither non-invasive (total effect HR 1.06, 95% CI 0.79–1.42) nor invasive (total effect HR 0.97, 95% CI 0.69–1.36) fertility treatments were associated with SARD.

**LIMITATIONS, REASONS FOR CAUTION:**

Exposure and outcome misclassification is possible as this study used published algorithms in health administrative data with unknown or imperfect sensitivity and specificity. Data on individual-level social and lifestyle factors and underlying causes of infertility were not available and thus were not included in the analysis.

**WIDER IMPLICATIONS OF THE FINDINGS:**

Infertility in the absence of fertility treatment may be an important risk marker for SARD in women who give birth. Greater health provider awareness of SARD symptoms and related gynaecological issues that may be present in women with infertility could facilitate earlier detection and treatment of SARD during the reproductive years.

**STUDY FUNDING/COMPETING INTERESTS(S):**

This research was funded by the Canadian Institutes of Health Research through a Banting Postdoctoral Fellowship to N.V.S. and Canada Research Chair to H.K.B. (2019-00158) and was supported by ICES, which is funded by an annual grant from the Ontario Ministry of Health and the Ministry of Long-Term Care. The analyses, conclusions, opinions, and statements expressed herein are solely those of the authors and do not reflect those of the funding organizations; no endorsement is intended or should be inferred. The funders had no role in considering the study design or in the collection, analysis, interpretation of data, writing of the report, or decision to submit the article for publication. M.Y.C. has consulted for Celltrion, Werfen, Organon, MitogenDx, AstraZeneca, Mallinckrodt Canada Inc, and Glaxo Smith Kline. All other authors have no conflicts of interest.

**TRIAL REGISTRATION NUMBER:**

N/A.

## Introduction

Approximately 12–25% of women experience infertility, defined as the inability to conceive after 12 months of unprotected sexual intercourse (Gurunath *et al.*, 2011). Aetiology is multi-factorial and can involve dysfunction in the endocrine and immune processes or anatomic structures that facilitate female reproduction (Strauss and Barbieri, 2014). There is growing interest in women’s long-term outcomes following infertility to guide sex-specific chronic disease prevention strategies. Initial studies showed that infertility is associated with increased future risk of reproductive cancers (Cetin *et al.*, 2008), with more recent studies reporting increased risks of cardiovascular diseases (Murugappan *et al.*, 2022), stroke (Liang *et al.*, 2022), and diabetes (Tobias *et al.*, 2015). The prevailing hypothesis is that infertility is a marker of underlying pathology which predisposes women towards these diseases, rather than a true causal factor in disease development (Luke *et al.*, 2016; Huttler *et al.*, 2023).

Systemic autoimmune rheumatic diseases (SARDs) include systemic lupus erythematosus, scleroderma/systemic sclerosis, Sjögren’s syndrome, and inflammatory myopathy. SARDs are characterized by an overactive immune response towards the body’s own tissues, resulting in progressive organ damage, disability, and premature mortality (Walsh and Rau, 2000; Thomas *et al.*, 2010). SARDs disproportionately affect females, with sex ratios ranging from 5:1 to 9:1 during early adulthood (Ngo *et al.*, 2014; Bairkdar *et al.*, 2021), which implicates the reproductive years as a potential window of susceptibility. Several biomedical studies have observed abnormal inflammation and autoantibodies in women with infertility compared to those without infertility (Vannuccini *et al.*, 2016; Deroux *et al.*, 2017), meriting a population-level assessment of differences in SARD incidence.

Epidemiological study of women’s long-term health following infertility is complex. First, many affected women use fertility treatments which often involve exogenous hormonal exposures that theoretically pose risks to maternal health distinct from that of underlying infertility (Smith *et al.*, 2021). Second, both infertility and its treatments are associated with adverse pregnancy outcomes (Messerlian *et al.*, 2013; Qin *et al.*, 2016; Chih *et al.*, 2021; Richmond *et al.*, 2022; Wang *et al.*, 2022). It is well-established that adverse pregnancy outcomes, namely preeclampsia, spontaneous preterm birth, and stillbirth, are each associated with a woman’s future risk of chronic disease (Rich-Edwards, 2009). Moreover, these three adverse pregnancy outcomes also appear to be associated with the onset of SARD (Scime *et al.*, 2023, 2024). Research on infertility and women’s long-term health has attempted to disentangle the effects of infertility from its treatments, but has not thoroughly accounted for the potential intermediate role of adverse pregnancy outcomes (Luke *et al.*, 2016; Huttler *et al.*, 2023).

This study investigated whether infertility, with or without receipt of fertility treatment, is associated with SARD among women who achieve a livebirth or stillbirth, accounting for the co-presence of adverse pregnancy outcomes around the time of that birth.

## Materials and methods

### Study design and setting

This population-based cohort study used linked administrative data covering all of ON, Canada. Ontario is Canada’s largest province, with 140 000 births per year and has a publicly funded health insurance plan that provides all medically necessary physician and hospital services at no direct cost to residents. We accessed and analysed data at ICES (Toronto), an independent, non-profit research institute whose legal status under Ontario’s health information privacy law allows it to collect and analyse health care and demographic data, without consent, for health system management, evaluation, and improvement.

### Ethical approval

The study was approved by the University of Toronto Ethics Board (no. 43489).

### Data sources

All hospital live births and stillbirths were identified in the MOMBABY dataset, which captures 98% of births in Ontario, deterministically linked using a unique encoded identifier with several ICES datasets. ICES data are valid, complete, and reliable (Williams and Young, 1996; Dunn *et al.*, 2011, 2019), and include information on perinatal characteristics (Better Outcomes Registry & Network), hospitalizations (Discharge Abstract Database), emergency department visits (National Ambulatory Care Reporting System), physician claims (Ontario Health Insurance Plan Claims), and sociodemographic factors (Registered Persons Database, Ontario Census Profiles).

### Study population

**Figure 1.**
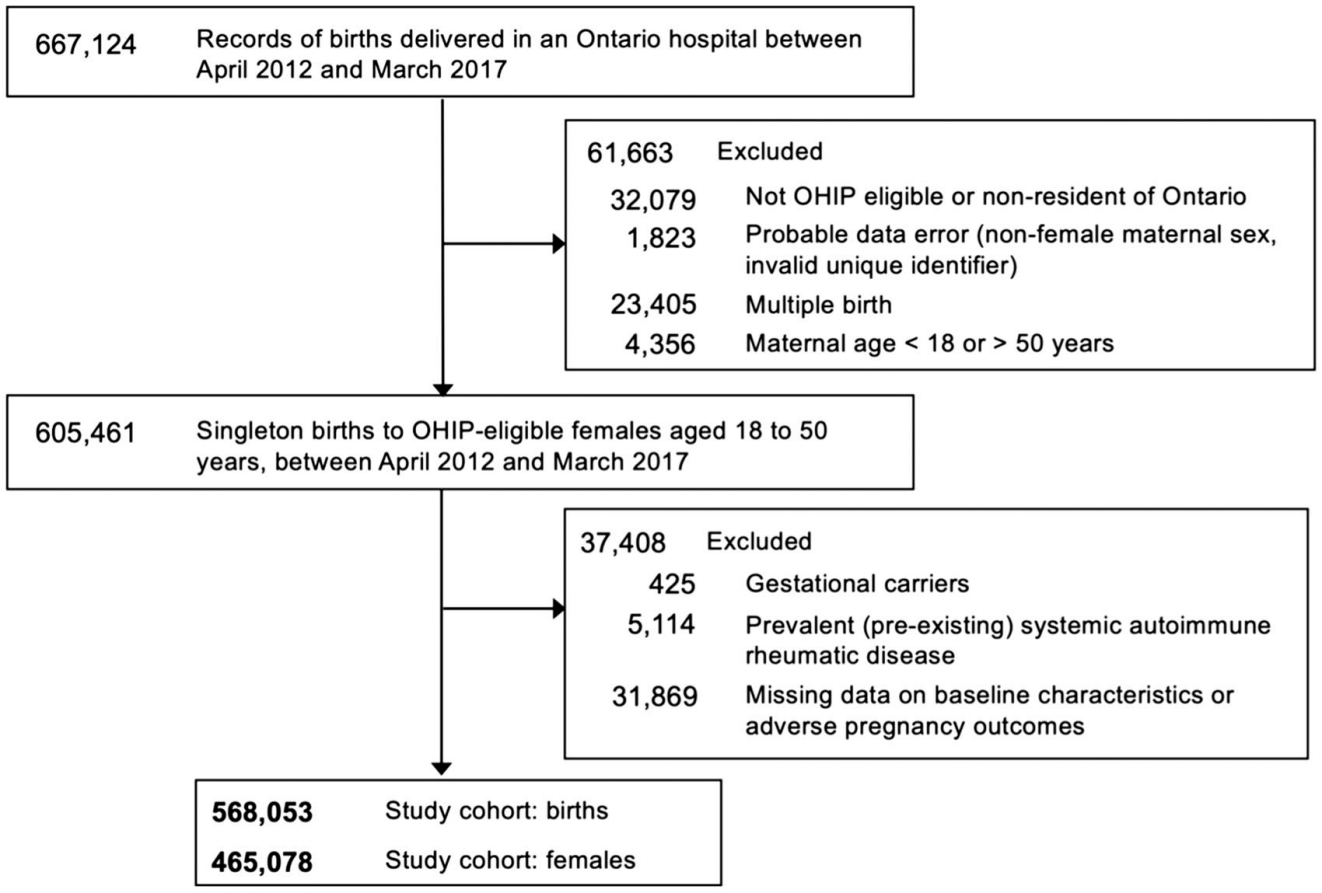
**Flow diagram of cohort creation.** OHIP, Ontario Health Insurance Plan.

The cohort included females aged 18–50 years with a singleton birth after 20 weeks gestational age (typically dated using first-trimester ultrasonography) (You *et al.*, 2010), with a delivery date between 1 April 2012 and 31 March 2017, and who were eligible for health insurance for at least 2 years before conception and up to delivery (Fig. 1). We excluded females who served as gestational carriers, who carried multi-foetal pregnancies to limit the influence of pregnancy and maternal risks distinctly associated with twins and higher order multiples, or who were previously diagnosed with SARD including undifferentiated connective tissue disease (a common precursor diagnosis before SARD is established). Women were followed to 31 March 2021, for a minimum of 4 years and a maximum of 9 years of observation.

### Exposure

Infertility was measured based on physician visits with International Classification of Diseases (ICD) diagnostic codes for infertility (ICD-9 628) in the 2 years prior to conception and receipt of fertility treatments recorded in the Better Outcomes Registry & Network, as done in prior population-based studies (Declercq *et al.*, 2014; Farland *et al.*, 2022a; Fine *et al.*, 2022). Women were classified as experiencing infertility with unassisted conception (i.e. diagnostic codes for infertility with no record of fertility treatment), infertility with assisted conception through non-invasive treatment (i.e. record of ovulation induction or intrauterine insemination), or infertility with assisted conception through invasive treatment (i.e. record of IVF or ICSI). Women without a history of health care for infertility and with unassisted conception were the reference group.

### Outcome

The primary outcome was SARD measured with diagnostic codes (ICD-9 710, ICD-10 M32.1, M32.8-M32.9, M33, M34, M35.0, M35.1, M35.8, M35.9, M36.0) using an administrative health data algorithm validated against medical records: by two physician visits with a SARD code, at least 2 months apart, but within a 2-year span; by one physician visit with a rheumatologist specialist with a SARD code; or by one hospitalization with a SARD diagnostic code (sensitivity >80%, specificity >70%) (Bernatsky *et al.*, 2011; Broten *et al.*, 2014).

### Covariates

Sociodemographic characteristics were age at delivery, neighbourhood income quintile, and rural residence based on health insurance registration and Census data linked with postal code. Pre-existing health conditions and behaviours were diabetes mellitus, chronic hypertension, obesity, endometriosis, and uterine fibroids measured using diagnostic codes on healthcare encounters within 2 years of conception, any other autoimmune disease measured using diagnostic codes on healthcare encounters from database inception to delivery (Supplementary Table S1) (Scime *et al.*, 2024), and maternal smoking documented on the delivery hospitalization record. Reproductive history included parity and pregnancy loss history. Adverse pregnancy outcomes were considered potential intermediate variables and included preeclampsia measured using diagnostic codes on healthcare encounters between conception and delivery, as well as stillbirth and spontaneous preterm birth (Harel *et al.*, 2020) measured using diagnostic and procedure codes on the delivery hospitalization record.

### Statistical analyses

We compared baseline characteristics of women in each infertility category using descriptive statistics and standardized differences (Austin, 2009). We estimated the cumulative incidence of SARD with 95% CIs in each exposure group. We then estimated hazard ratios (HR) and 95% CIs for the association between infertility and incidence of SARD following birth using marginal structural Cox proportional hazards models with stabilized inverse probability weights (Robins *et al.*, 2000; Vanderweele, 2009). Person-time at risk was counted in days from the date of delivery to the date of SARD diagnosis or censoring at death, loss of OHIP eligibility, or end of the study period, whichever came first. A robust sandwich estimator of the variance was used to account for more than one birth by the same woman during the study period (Crowther and Lambert, 2014). The proportional hazards assumption was assessed and confirmed using smoothed plots and statistical tests of Schoenfeld residuals and time-by-covariate interactions.

First, we estimated the total effect between infertility and SARD that adjusted for confounding variables using inverse probability of exposure weights. To calculate the exposure weights, we fit a polytomous logistic regression model for infertility (with no infertility/unassisted conception as the referent outcome group) conditional on the following baseline characteristics: maternal age, income quintile, rurality, diabetes, hypertension, obesity, other autoimmune disease, and smoking. Second, we estimated the controlled direct effect between infertility and SARD that additionally accounted for intermediate variables using the product of the inverse probability of exposure weights and three intermediate variable weights. To calculate the intermediate variable weights, we fit separate logistic regression models for preeclampsia, spontaneous preterm birth, and stillbirth conditional on infertility and baseline characteristics. Direct effects were only estimated in the presence of a possible exposure–outcome association, wherein the total effect HR ≥1.10 (regardless of 95% CI to limit reliance on statistical significance). All inverse probability weights were truncated at the 99th percentile to improve model precision.

We conducted five additional analyses. First, we explored effect heterogeneity by stratifying the models on parity (primiparous, multiparous) and pregnancy loss history (0, 1 or more miscarriage), separately. Reproductive history was considered in this separate step to avoid inducing bias in the marginal structural models (Howards *et al.*, 2007; Huttler *et al.*, 2023), given the complex biopsychosocial influences between parity, pregnancy loss history, infertility, pursuit of infertility treatment, continued conception attempts until achieving a birth (on which cohort selection was conditioned), and other maternal characteristics (Farr *et al.*, 2009; Pearson *et al.*, 2009). Second, we used a more stringent definition for the infertility with treatment groups to additionally require a healthcare visit for infertility in the 2 years prior to conception. Third, we restricted the models to women aged <38 years at delivery to minimize the potential for typical age-related declines in fertility to influence our results (American College of Obstetricians and Gynecologists Committee on Gynecologic Practice and the Practice Committee of the American Society for Reproductive Medicine, 2014). Fourth, we restricted the models to women without endometriosis or uterine fibroids to determine whether results were attenuated after removing gynaecological conditions that may result in infertility, adverse pregnancy outcomes, and chronic health conditions (Kvaskoff *et al.*, 2015; Farland *et al.*, 2022b; Velez *et al.*, 2022). Fifth, given the high co-occurrence among autoimmune diseases (Conrad *et al.*, 2023), we excluded females with any pre-existing autoimmune disease other than SARD to reduce the potential influence of clinical surveillance bias on SARD diagnoses (Kolanska *et al.*, 2021).

## Results

The cohort included 568 053 singleton births to 465 078 females (Fig. 1). Overall, 9.2% of births were to females with infertility who conceived without treatment, 1.4% were to females who conceived with non-invasive fertility treatment, and 1.4% were to females who conceived with invasive fertility treatment. Compared to females without infertility, females with infertility with or without fertility treatment were more often older in age, were from a higher neighbourhood income quintile, lived in a non-rural area, and were non-smokers (Table 1). Females with infertility were also more likely to be primiparous and report at least one prior pregnancy loss, and to experience an adverse pregnancy outcome during the index birth.

**Table 1.** Baseline characteristics by infertility status (N = 568 053 births).

	No infertility or treatment	Infertility, no treatment	Non-invasive infertility treatment	Invasive infertility treatment
	N = 499 756	N = 52 047	N = 8136	N = 8114
Proportion of the cohort	88.0	9.2	1.4	1.4
Maternal age, years				
<25	72 920 (14.6)	1639 (3.1)*	189 (2.3)*	29 (0.4)*
25–34	326 331 (65.3)	30 747 (59.1)*	4802 (59.0)*	3264 (40.2)*
35–44	99 875 (20.0)	19 492 (37.5)*	3119 (38.3)*	4432 (54.6)*
≥45	630 (0.1)	169 (0.3)	26 (0.3)	389 (4.8)*
Neighbourhood income quintile				
Q1 (lowest)	111 636 (22.3)	9008 (17.3)*	1085 (13.3)*	791 (9.7)*
Q2	101 087 (20.2)	9625 (18.5)	1389 (17.1)	1239 (15.3)*
Q3	103 168 (20.6)	10 976 (21.1)	1769 (21.7)	1674 (20.6)
Q4	104 058 (20.8)	12 378 (23.8)	2096 (25.8)*	2183 (26.9)*
Q5 (highest)	79 807 (16.0)	10 060 (19.3)	1797 (22.1)*	2227 (27.4)*
Rural residence	54 470 (10.9)	3378 (6.5)*	647 (8.0)*	422 (5.2)*
Comorbidities				
Diabetes	10 430 (2.1)	1761 (3.4)	306 (3.8)	220 (2.7)
Hypertension	2499 (0.5)	365 (0.7)	68 (0.8)	53 (0.7)
Obesity	85 144 (17.0)	9948 (19.1)	2145 (26.4)*	1250 (15.4)
Autoimmune disease	8878 (1.8)	1201 (2.3)	202 (2.5)	212 (2.6)
Endometriosis	1828 (0.4)	725 (1.4)*	78 (1.0)	225 (2.8)*
Uterine fibroids	1550 (0.3)	701 (1.3)*	65 (0.8)	122 (1.5)*
Maternal smoking	58 512 (11.7)	2168 (4.2)*	281 (3.5)*	114 (1.4)*
Primiparous	205 007 (41.0)	25 749 (49.5)*	5261 (64.7)*	5456 (67.2)*
Number of prior pregnancy losses				
0	382 968 (76.6)	32 714 (62.9)*	5797 (71.3)*	5579 (68.8)*
1	85 377 (17.1)	10 739 (20.6)	1481 (18.2)	1546 (19.1)
≥2	31 411 (6.3)	8594 (16.5)*	858 (10.5)*	989 (12.2)*
Pre-eclampsia	22 277 (4.5)	2862 (5.5)	620 (7.6)*	606 (7.5)*
Spontaneous preterm birth	21 888 (4.4)	2773 (5.3)	412 (5.1)	545 (6.7)*
Stillbirth	1250 (0.3)	127 (0.2)	25 (0.3)	35 (0.4)

All data are presented as a number (%).

* Indicates an important standardized difference >0.10 when compared to women in the no infertility or treatment group.

At 9 years following birth (median = 6.5 years of follow-up per birth [interquartile range = 5.2–7.7]), the incidence of SARD per 10 000 person-years was 9.3 in females without infertility, 12.5 in females with infertility without treatment, 10.9 in females with non-invasive fertility treatment, and 10.9 in females with invasive fertility treatment (Table 2). The total effects model adjusting for baseline characteristics indicated an elevated risk of SARD in females with infertility without treatment (HR 1.25, 95% CI 1.12–1.40), but similar risks of SARD in females with non-invasive (HR 1.06, 95% CI 0.79–1.42) and invasive fertility treatment (HR 0.97, 95% CI 0.69–1.36), compared to those without infertility. The association between infertility without treatment and elevated risk of SARD was unchanged in the direct effects model that additionally accounted for the intermediate role of adverse pregnancy outcomes.

**Table 2.** Association between infertility and incident onset of systemic autoimmune rheumatic disease after childbirth.

Exposure	No. at risk	Person-years of follow-up	No. with SARD	Incidence per 10 000 person-years (95% CI)	Hazard ratio (95% CI)		
					Crude effect	Total effect	Direct effect
No infertility or treatment (ref)	499 756	3 207 873.5	2983	9.3 (9.0, 9.6)	1.00	1.00	1.00
Infertility, no treatment	52 047	332 841.9	417	12.5 (11.4, 13.8)	1.35 (1.22, 1.49)	1.25 (1.12, 1.40)	1.25 (1.12, 1.40)
Non-invasive infertility treatment	8136	51 516.8	56	10.9 (8.4, 14.1)	1.17 (0.90, 1.53)	1.06 (0.79, 1.42)	
Invasive infertility treatment	8114	51 182.6	56	10.9 (8.4, 14.2)	1.18 (0.91, 1.54)	0.97 (0.69, 1.36)	

Total effect models adjusted for potential confounding from baseline characteristics: maternal age, income quintile, rurality, diabetes, hypertension, obesity, and smoking. Direct effect models were only estimated when the total effect ≥1.10 (indicating a small positive association or stronger) and accounted for potential intermediate variables: preeclampsia, spontaneous preterm birth, and stillbirth.

SARD, systemic autoimmune rheumatic disease.

The results were robust in additional analyses restricting to females who were primiparous, reported no prior pregnancy loss, received fertility treatment specifically for clinically recognized infertility, aged <38 years, or had no diagnosis of endometriosis, uterine fibroids, or other autoimmune diseases (Table 3). Of note, total effect point estimates were larger for non-invasive fertility treatment in multiparous females (HR 1.26, 95% CI 0.77–2.05) and for non-invasive (HR 1.30, 95% CI 0.83–2.03) and invasive (HR 1.22, 95% CI 0.71–2.10) fertility treatment in females with ≥1 prior pregnancy loss, and persisted in magnitude in direct effect models; however the 95% CI were wide and included the null (Table 3).

**Table 3.** Association between infertility and incident onset of systemic autoimmune rheumatic disease after childbirth, further assessed by parity, prior pregnancy loss, case definition for fertility treatments, maternal age <38 years, absence of endometriosis or uterine fibroids, and absence of existing autoimmune disease.

Exposure	No. at risk	Person-years of follow-up	No. with SARD	Incidence per 10 000 person-years (95% CI)	Hazard ratio (95% CI)		
					Crude effect	Total effect	Direct effect
Primiparous							
No infertility or treatment (ref)	205 007	1 317 807.3	1205	9.1 (8.6, 9.7)	1.00 (ref)	1.00 (ref)	1.00 (ref)
Infertility, no treatment	25 749	164 646.7	202	12.3 (10.7, 14.1)	1.34 (1.16, 1.56)	1.24 (1.05, 1.46)	1.25 (1.06, 1.48)
Non-invasive infertility treatment	5261	33 296.5	37	11.1 (8.1, 15.3)	1.22 (0.88, 1.69)	0.90 (0.62, 1.32)	
Invasive infertility treatment	5456	34 565.5	36	10.4 (7.5, 14.4)	1.14 (0.82, 1.59)	0.95 (0.59, 1.52)	
Multiparous							
No infertility or treatment (ref)	294 749	1 890 066.2	1778	9.4 (9.0, 9.9)	1.00 (ref)	1.00 (ref)	1.00 (ref)
Infertility, no treatment	26 298	168 195.2	215	12.8 (11.2, 14.6)	1.36 (1.18, 1.57)	1.26 (1.08, 1.47)	1.24 (1.07, 1.44)
Non-invasive infertility treatment	2875	18 220.4	19	10.4 (6.7, 16.3)	1.11 (0.71, 1.75)	1.26 (0.77, 2.05)	1.23 (0.75, 2.01)
Invasive infertility treatment	2658	16 617.2	20	12.0 (7.8, 18.7)	1.28 (0.83, 2.00)	1.03 (0.57, 1.86)	
No prior pregnancy losses							
No infertility or treatment (ref)	382 968	2 460 183.0	2216	9.0 (8.6, 9.4)	1.00 (ref)	1.00 (ref)	1.00 (ref)
Infertility, no treatment	32 714	209 090.0	245	11.7 (10.3, 13.3)	1.30 (1.14, 1.49)	1.24 (1.08, 1.43)	1.24 (1.08, 1.43)
Non-invasive infertility treatment	5797	36 675.8	33	9.0 (6.4, 12.7)	1.00 (0.71, 1.41)	0.96 (0.65, 1.40)	
Invasive infertility treatment	5579	35 302.1	37	10.5 (7.6, 14.5)	1.17 (0.84, 1.61)	0.86 (0.56, 1.31)	
≥1 prior pregnancy losses							
No infertility or treatment (ref)	116 788	747 690.5	767	10.3 (9.6, 11.0)	1.00 (ref)	1.00 (ref)	1.00 (ref)
Infertility, no treatment	19 333	123 751.9	172	13.9 (12.0, 16.1)	1.36 (1.15, 1.60)	1.22 (1.02, 1.45)	1.22 (1.02, 1.45)
Non-invasive infertility treatment	2339	14 841.0	23	15.5 (10.3, 23.3)	1.51 (1.00, 2.29)	1.30 (0.83, 2.03)	1.27 (0.81, 2.00)
Invasive infertility treatment	2535	15 880.6	19	12.0 (7.6, 18.8)	1.17 (0.74, 1.84)	1.22 (0.71, 2.10)	1.10 (0.63, 1.93)
Stringent case definition for fertility treatment ^a^							
No infertility or treatment (ref)	499 756	3 207 874	2985	9.3 (9.0, 9.6)	1.00 (ref)	1.00 (ref)	1.00 (ref)
Infertility, no treatment	52 047	332 842	417	12.5 (11.4, 13.8)	1.35 (1.22, 1.49)	1.25 (1.12, 1.40)	1.25 (1.12, 1.40)
Non-invasive infertility treatment	7333	46 370	53	11.4 (8.7, 15.0)	1.23 (0.94, 1.62)	1.14 (0.85, 1.55)	1.12 (0.82, 1.51)
Invasive infertility treatment	7834	49 431	56	11.3 (8.7, 14.7)	1.22 (0.94, 1.59)	1.01 (0.72, 1.42)	
Maternal age <38 years							
No infertility or treatment (ref)	462 276	2 968 108.1	2720	9.2 (8.8, 9.5)	1.00 (ref)	1.00 (ref)	1.00 (ref)
Infertility, no treatment	43 001	274 798.1	327	11.9 (10.7, 13.3)	1.30 (1.16, 1.46)	1.24 (1.09, 1.39)	1.23 (1.09, 1.39)
Non-invasive infertility treatment	6679	42 247.1	43	10.2 (7.5, 13.7)	1.11 (0.82, 1.51)	1.04 (0.75, 1.44)	
Invasive infertility treatment	5309	33 433.3	32	9.6 (6.8, 13.5)	1.05 (0.74, 1.49)	0.91 (0.60, 1.36)	
No endometriosis or fibroids							
No infertility or treatment (ref)	496 405	3 186 338.0	2948	9.3 (8.9, 9.6)	1.00 (ref)	1.00 (ref)	1.00 (ref)
Infertility, no treatment	50 658	323 973.3	405	12.5 (11.3, 13.8)	1.35 (1.22, 1.50)	1.25 (1.12, 1.40)	1.25 (1.12, 1.40)
Non-invasive infertility treatment	7994	50 625.4	55	10.9 (8.3, 14.2)	1.18 (0.90, 1.54)	1.07 (0.80, 1.44)	
Invasive infertility treatment	7786	49 084.7	56	11.4 (8.8, 14.8)	1.24 (0.95, 1.61)	1.02 (0.73, 1.43)	
No existing autoimmune disease							
No infertility or treatment (ref)	490 867	3 151 666.0	2786	8.8 (8.5, 9.2)	1.00 (ref)	1.00 (ref)	1.00 (ref)
Infertility, no treatment	50 842	325 214.7	394	12.1 (11.0, 13.4)	1.37 (1.23, 1.52)	1.27 (1.14, 1.43)	1.27 (1.14, 1.43)
Non-invasive infertility treatment	7932	50 240.8	53	10.5 (8.1, 13.8)	1.20 (0.91, 1.57)	1.10 (0.82, 1.48)	1.08 (0.80, 1.45)
Invasive infertility treatment	7902	49 862.9	49	9.8 (7.4, 13.0)	1.11 (0.84, 1.48)	0.95 (0.67, 1.36)	

Total effect models controlled for potential confounding from baseline characteristics: maternal age, income quintile, rurality, diabetes, hypertension, obesity, and smoking. Direct effect models were only estimated when the total effect ≥1.10 (indicating a small positive association or stronger) and accounted for potential intermediate variables: preeclampsia, spontaneous preterm birth, and stillbirth.

a The more stringent case definition for fertility treatment required both receipt of fertility treatments and a healthcare visit for infertility in the 2 years prior to conception.

SARD, systemic autoimmune rheumatic disease.

## Discussion

In this population-based cohort study of women who gave birth, those who experienced infertility but did not use fertility treatment had a higher incidence of SARD up to 9 years after delivery than those who did not experience infertility, even after accounting for their higher rates of preeclampsia, spontaneous preterm birth, and stillbirth. Interestingly, females who used any fertility treatment had a similar risk of SARD to those without infertility with some exceptions by subgroup, although the interpretation was less conclusive because of imprecise estimates. These data suggest that infertility as a health condition itself, independent of fertility treatments and adverse pregnancy outcomes, may be a risk marker for SARD in reproductive-aged women.

To our knowledge, only three case-control studies have examined the association between infertility and subsequent risk of SARD in women. One study found that 7.2% of women with scleroderma reported infertility before diagnosis compared to 3.5% of community-based, age-matched controls, with an adjusted odds ratio of 2.1 (95% CI 0.6–7.8) after controlling for smoking and sociodemographic factors (Englert *et al.*, 1992). Another found that 7.8% of women with scleroderma reported infertility before diagnosis compared to 2.6% of family or friend controls, with an unadjusted risk ratio of 3.0 (95% CI 0.8–11.1) (Silman and Black, 1988). In contrast, a third study found that 4.9% of women with incident systemic lupus erythematosus reported infertility compared to 6.1% of hospital-based controls (Grimes *et al.*, 1985). These prior works are foremost limited by potential recall bias, confounding, and low statistical power. Our study advances this literature as the first to use a population-based and longitudinal cohort design to show an association between infertility without treatment and elevated incidence of SARD in women who achieve a birth. Importantly, this association persisted after accounting for sociodemographic factors, comorbidities, reproductive history and adverse pregnancy outcomes, and spanned nearly a decade after birth.

Mounting evidence supports the interpretation that infertility could be an initial manifestation of subclinical SARD in reproductive-aged women. In a clinical study of 136 women with infertility, 1.5% were found to have undiagnosed systemic lupus erythematosus following medical history and laboratory investigations; this yielded an estimate of 1470 cases per 100 000 females with infertility compared to 50–90 undiagnosed cases per 100 000 females in the general population (Geva *et al.*, 2004). The association between nulliparity and increased risk of SARD reported in some, but not all, studies (Marder and Somers, 2014) is thought to be partly attributable to subclinical SARD impairing fertility and consequently reducing parity (Lambe *et al.*, 2004; Ulff-Møller *et al.*, 2009). Ovulatory dysfunction and ovarian depletion are frequent causes of infertility (Carson and Kallen, 2021) and potentially share pathophysiological mechanisms with infertility and SARD. The inflammatory cascade of SARD can suppress the hypothalamic–pituitary–ovarian axis. For example, treatment-naïve women with SARD have lower gonadotropin-releasing hormone levels and higher prolactin levels, and report higher rates of menstrual irregularities than women without SARD (Pasoto *et al.*, 2002; Haga *et al.*, 2005; Shabanova *et al.*, 2008; Lu *et al.*, 2016). Ovarian reserve is diminished even in the presence of mild SARD, possibly due to an autoimmune response targeting the ovaries (Silva *et al.*, 2014). Sexual dysfunction and neuropsychiatric symptoms are additional possible mechanisms linking infertility and SARD. Women with SARD frequently report genitourinary symptoms, pain, and sexual impairment that could present prodromally (Minopoulou *et al.*, 2023), and experience higher rates of headache and mood disorders (Unterman *et al.*, 2011; Amaral *et al.*, 2013). These circumstances may reduce the frequency of sexual intercourse and therefore fertility attempts. Given the multi-year prodromal phase of SARD, it is conceivable that these signs of ovulatory, ovarian, sexual, and neuropsychiatric dysfunction affect women before a clinical disease is diagnosed.

Endometriosis, antiphospholipid syndrome, and age-related ovarian depletion are additional causes of infertility that could overlap with SARD. Endometriosis is an inflammatory disease accompanied by immune system dysregulation, which is thought to explain why endometriosis is frequently comorbid with SARD and other autoimmune diseases (Shigesi *et al.*, 2019) and why some experts believe that endometriosis may be an autoimmune disease itself. Both SARD and infertility increase in prevalence as women age (Conrad *et al.*, 2023). Notwithstanding, our results were robust to the exclusion of women with endometriosis and of older age, separately. Antiphospholipid syndrome, characterized by the presence of anticardiolipin antibodies or lupus anticoagulant, is a rare condition that often co-exists with fertility issues and SARD (Schreiber *et al.*, 2018). Antiphospholipid syndrome is often incompletely tested in clinical practice and suboptimally coded in health administrative data (Egiziano *et al.*, 2020; Ballif *et al.*, 2023), and was thus not fully captured in our sensitivity analysis excluding women with other autoimmune diseases. Additional research exploring the clinical relevance of antiphospholipid antibodies in the association between infertility and SARD is needed.

The lack of association between infertility with treatment and SARD, as evidenced by total effect point estimates near the null, could be explained by systematic differences in underlying infertility causes and patient factors. Advanced age, tubal, and male factor causes of infertility are over-represented in those who seek fertility treatments (Carson and Kallen, 2021), yet have little plausible biological relation to female SARD or autoimmunity. Women who are socioeconomically advantaged and of White race are more likely to access fertility treatments (Jain, 2006), yet conversely experience the lowest rates of SARD (Roberts and Erdei, 2020; Conrad *et al.*, 2023). This ‘healthy patient’ effect has been noted in prior studies which also found greater risks of adverse health outcomes (e.g. mortality (Murugappan *et al.*, 2021), certain cancers (Yli-Kuha *et al.*, 2012)) in women with infertility, but similar risks in women who use fertility treatment, compared to women without infertility. Detection of SARD during a preconceptional diagnostic assessment of infertility is also possible (Kallen and Arici, 2003), and these cases would have been excluded from our study of post-delivery incidence. Notwithstanding, wide CIs spanning a medium protective to the large harmful effect of infertility with treatment on incident SARD limits the certainty of these interpretations.

Strengths of this study include the large sample size and population-based approach, validated outcome definition for SARD, and use of advanced modelling techniques to simultaneously account for several confounding and intermediate factors; however, there are limitations to consider. Exposure misclassification is possible. Women who experienced infertility without treatment may have been misclassified in the reference group if they did not discuss this experience with a healthcare provider. Women with repeated pregnancy loss may have been misclassified as having infertility without treatment, as varying clinical definitions for infertility (i.e. inability to achieve conception versus inability to achieve a successful pregnancy) (Zegers-Hochschild *et al.*, 2017; Practice Committee of the American Society for Reproductive Medicine, 2020) may have led to diagnostic codes for infertility recorded in their care encounters. Our data suggest this is possible to a small degree given the slightly higher proportion of two or more pregnancy losses (16.5%) in the infertility without treatment group compared to the fertility treatment groups (10–12%), and similar total effect point estimates (despite wide 95% CI) across all infertility groups when the analysis was restricted to females with one or more prior pregnancy loss. Sensitivity and specificity of the validated algorithm for SARD are suitable for population-level epidemiologic research, but the algorithm is imperfect and thus some cases may have been missed (Bernatsky *et al.*, 2011; Broten *et al.*, 2014). We lacked data on individual-level sociodemographic characteristics (e.g. race, education) and possible lifestyle (e.g. alcohol use) and reproductive (e.g. age at menarche) risk factors shared by infertility and SARD and were thus unable to include these potential confounding factors in our analysis. We also lacked data on underlying causes of infertility; the distribution of causes likely differed in those with versus without fertility treatment, which may have an important explanatory role in the different associations with SARD that we observed by treatment status. The rarity of both infertility with treatment and SARD diminished the precision of estimates and thus interpretability for some models. Finally, these findings are not generalizable to nulliparous women, as they were not included in this study dataset based on obstetric births.

SARDs are rare chronic diseases that can result in progressive disability and irreversible organ damage if not promptly diagnosed and treated (Steen and Medsger, 2000; Kernder *et al.*, 2021). Findings from this study suggest that fertility care presents an opportunity to carefully screen women experiencing infertility for rheumatic symptoms including overlapping gynaecological symptoms and, if indicated, start investigations or consider referral to rheumatology as appropriate. This study, along with the growing body of evidence on long-term chronic disease risk following female infertility (Huttler *et al.*, 2023), also supports a potential role for patient counselling on the broader health impacts of an infertility diagnosis to ensure women seek timely care for disease signs and symptoms as they age. Additional research that examines the incidence of SARD by infertility cause and pinpoints the shared pathophysiology between infertility and SARD would be valuable for guiding risk stratification efforts.

## Conclusion

In this population-based cohort study of women who gave birth in Ontario, a history of infertility without fertility treatment was associated with a higher future risk of SARD, independent of co-existing adverse pregnancy outcomes. Future research efforts should seek to corroborate this association by infertility cause, with a focus on possible mechanisms related to ovulatory, ovarian, and sexual dysfunction. Greater health provider awareness of SARD symptoms and related gynaecological issues that may present in women with infertility could facilitate earlier detection and treatment of SARD during the reproductive years.

## Supplementary Material

deae253_Supplementary_Table_S1

## Data Availability

The dataset from this study is held securely in coded form at ICES. While data-sharing agreements prohibit ICES from making the dataset publicly available, access might be granted to those who meet prespecified criteria for confidential access, available at www.ices.on.ca/DAS.
