# Supplementary material for: Association between infertility and incident onset of systemic autoimmune rheumatic disease after childbirth: a population-based cohort study
Source: Hum Reprod. 2024 Dec 5;40(1):157–66. doi: 10.1093/humrep/deae253 (PMC11700898; doi:10.1093/humrep/deae253)
Supplement: deae253_Supplementary_Table_S1 [file deae253_supplementary_table_s1.pdf]

OXFORD
human reproduction

Human Reproduction, 2024, 1–1
https://doi.org/10.1093/humrep/deae253
Supplementary Data

Supplementary Table S1. Diagnostic codes and validated algorithms used to measure other autoimmune diseases (study covariate), excluding the primary outcome of SARD.

	Acute care		Acute and outpatient care
**Autoimmune disease**	**ICD-9 codes**	**ICD-10-CA codes**	**Validated algorithm**
Addison’s disease	255.4	E27.1	None
Ankylosing spondylitis	720.0	M45	2 physician visits (OHIP: 720) with one or more by a specialist (rheumatologist, internal medicine) or one acute care visit (ICD-9: 720.0; ICD-10: M45) in a 2-year period
Autoimmune haemolytic anaemia	283.0	D59.1	None
Autoimmune hepatitis	NA	K75.4	None
Celiac disease	579.0	K90.0	2 physician visits (OHIP: 579) or one acute care visit (ICD-9: 579; ICD-10: K90) in a 3-year period
Crohn’s disease	555	K50	Inflammatory bowel disease: 5 physician visits (OHIP: 555, 556) or acute care visits (ICD-9: 555, 556; ICD-10: K50, K51) in a 4-year period
Guillain Barre syndrome	357.0	G61.0	None
Hashimoto’s thyroiditis	245.2	E06.3	None
Idiopathic thrombocytopenic purpura	NA	D69.3	None
Multiple sclerosis	340	G35	5 physician visits (OHIP: 340) or 1 acute care visit (ICD-9: 340; ICD-10: G35) in a 2-year period
Myasthenia gravis	358.0	G70.0	None
Pernicious anaemia	281.0	D51.0	None
Polymyalgia rheumatica	725	M35.3	None
Primary biliary cirrhosis	571.6	K74.3	None
Psoriatic arthritis	696.0	L40.5, M07.0, M07.1, M07.2, M07.3, M09.0	3 physician visits (OHIP: 720, 721) with one or more by a specialist (rheumatologist, internal medicine) and 1 physician visit (OHIP: 696) with a psoriasis code or 1 acute care visit (ICD-9: 696.0; ICD-10: L40.5, M07.0, M07.1, M07.2, M07.3, M09.0)
Rheumatoid arthritis	714.0-714.2, 714.9	M05, M06	3 physician visits (OHIP: 714) with 1 or more by a specialist (rheumatologist, internal medicine, orthopaedic surgeon) or 1 acute care visit (ICD-9: 714; ICD-10: M05, M06) in a 2-year period
Sarcoidosis	135, 321.4	D86, G53.2, M63.3	2 physician visits (OHIP: 135) or acute care visits (ICD-9: 135, 321.4; ICD-10: D86, G53.2, M63.3) at least 2 weeks apart, in 2 years
Systemic vasculitis	446	M30, M31	None
Thyrotoxicosis/Grave’s disease	242.0	E05.0	None
Ulcerative colitis	556	K51	Inflammatory bowel disease: 5 physician visits (OHIP: 555, 556) or acute care visits (ICD-9: 555, 556; ICD-10: K50, K51) in a 4-year period

Acute care: measured by diagnostic codes in ≥1 hospitalizations or emergency department visits after the birth, since physician visit codes contain three digits and are too broad to distinguish many autoimmune diseases.
Acute and outpatient care: algorithms validated against medical records that use diagnostic codes recorded in outpatient physician visits, hospitalizations, and emergency department visits.
SARD, systemic autoimmune rheumatic disease; OHIP, Ontario Health Insurance Plan; NA, not applicable as there is no 4-digit ICD-9 code specific enough for the condition.

© The Author(s) 2024. Published by Oxford University Press on behalf of European Society of Human Reproduction and Embryology.
This is an Open Access article distributed under the terms of the Creative Commons Attribution License (https://creativecommons.org/licenses/by/4.0/), which permits unrestricted reuse, distribution, and reproduction in any medium, provided the original work is properly cited.
Human Reproduction, 2024, 00, 1–11
https://doi.org/10.1093/humrep/deae253
Original Article
